# Impact of Diets Including Agro-Industrial By-Products on the Fatty Acid and Sterol Profiles of Larvae Biomass from *Ephestia kuehniella*, *Tenebrio molitor* and *Hermetia illucens*

**DOI:** 10.3390/insects12080672

**Published:** 2021-07-26

**Authors:** Fatma Boukid, Jordi Riudavets, Lidia del Arco, Massimo Castellari

**Affiliations:** 1 Food Safety and Functionality Programme, Institute of Agriculture and Food Research and Technology (IRTA), 17121 Monells, Spain; massimo.castellari@irta.cat; 2 Sustainable Plant Protection Programme, Institute of Agriculture and Food Research and Technology (IRTA), Carretera de Cabrils, km 2, 08348 Cabrils, Spain; jordi.riudavets@irta.cat (J.R.); lidia.delarco@irta.cat (L.d.A.)

**Keywords:** insect, diet, fatty acids, cholesterol, phytosterols, circular economy

## Abstract

**Simple Summary:**

Insects are a promising source of lipids. Their fatty acid compositions can vary as a function of diet composition, rearing conditions and developmental stage. In the present study, different agro-industrial by-products were used to feed the insects. Then, the fatty acids and sterols were determined. Notably, these profiles were assessed for the first time for *E. kuehniella*. According to our results, fatty acid profiles showed differences depending on diet composition, but mostly depended on species. Sterols varied significantly as a function of diet composition and species, showing low cholesterol and high campesterol and β-sitosterol levels in *H. illucens*, and high cholesterol and low campesterol contents in *T. molitor* and *E. kuehniella*. These results suggest that insects are an interesting alternative source of fat for humans and animals, which might promote the use of insects for circular economy practices.

**Abstract:**

Rearing insects on agro-industrial by-products is a sustainable strategy for the circular economy while producing valuable products for feed and foods. In this context, this study investigated the impact of larvae diet containing agro-industrial by-products on the contents of fatty acids and sterols of *Ephestia kuehniella* (Zeller) (Lepidoptera: Pyralidae), *Tenebrio molitor* (L.) (Coleoptera: Tenebrionidae), and *Hermetia illucens* (L.) (Diptera: Stratiomyidae). For each insect, selected diets were formulated using single or combined agro-industrial by-products (i.e., apricot, brewer’s spent grain and yeast, and feed mill) and compared to a control diet. Fatty acid profiles showed differences depending on diet composition, but mostly depended on species: *H. illucens* was characterized by the abundance of C12:0, C16:0 and C18:2, whereas C:16, C18:1(n-9c), and C18:2(n-6c) were predominant in *T. molitor* and *E. kuehniella*. Sterols significantly varied as a function of diet composition and species. *H. illucens* showed low cholesterol levels and high campesterol and β sitosterol levels (0.031, 0.554 and 1.035 mg/g, respectively), whereas *T. molitor* and *E. kuehniella* had high cholesterol and low campesterol contents (1.037 and 0.078 g/kg, respectively, for *T. molitor*; 0.873 and 0.132 g/kg, respectively, for *E. kuehniella*).

## 1. Introduction

Waste management and by-product valorization represent key elements for ensuring resource efficiency and sustainability. Currently, the agro-industrial sector generates large amounts of wastes (e.g., post-harvest losses) and processing by-products, representing a significant disposal problem for the industry [1]. In the EU, the total amount of agro-industrial by-products is estimated to be around 16 million tons, where the top producers are Germany (3 million tons), the United Kingdom (2.6 million tons), Italy (1.9 million tons), France (1.8 million tons), and Spain (1.6 million tons) [2]. Notably, these by-products are rich sources of bioactive compounds [3,4]. Therefore, developing eco-friendly solutions for their reuse and recycling is essential from economic and environmental perspectives.

Insects can be a green alternative technology for the bioconversion of agro-industrial residues (e.g., fruit and vegetable wastes, food wastes, animal by-products, and manure) and their repurposing for animal feed, compost, and biofuels [5,6,7,8]. Rearing insects requires less land, water, and space compared to other livestock productions; therefore, it can be described as more sustainable than conventional food sources [9,10]. Larvae of *Hermetia illucens* (L.) (Diptera: Stratiomyidae) (black soldier flies) and *Tenebrio molitor* (L.) (Coleoptera: Tenebrionidae) (yellow mealworm) are two of the most interesting insect species for food and feed applications [11,12,13,14]. Recently, *T. molitor* was included in a list of edible insect species authorized as novel foods in the EU [15].

Insects are a promising source of lipids, where the fatty acid composition is closely related to diet composition, rearing conditions and the developmental stage [8,16,17,18,19,20,21]. Research focused on *H. illucens* and *T. molitor* indicated that the inclusion of by-products such as wheat bran, brewery spent grains, bread and cookie leftovers or winery waste in their diet can modify the fatty acid profile of the larval biomass [22,23,24].

Recently, we investigated the bioconversion of agro-industrial by-products with three different insect species (i.e., *Ephestia kuehniella* (Zeller) (Lepidoptera: Pyralidae), *T. molitor*, and *H. illucens*) to develop an efficient waste management strategy [25]. This study included the assessment of 26 diets containing different agro-industrial by-products (non-marketable apricots, brewer’s spent grains, brewer’s spent yeast, feed mill byproducts including broken cereal grains, and hatchery waste including eggshell debris, fluff, infertile eggs, dead embryos, and egg fluids) or mixtures thereof. The main reason behind the selection of these by-products is their high relevance at regional level and the interest of the industry requiring innovative ways for their valorization. In this study, we identified several diets, based on specific by-products, which improved the growth performance for each species compared to standard diets.

The aim of present study was, therefore, to further investigate the effect of these selected diets, formulated with different agro-industrial by-products, on the fatty acids and sterols composition of the larval biomass of *E. kuehniella*, *T. molitor*, and *H. illucens* in comparison with a standard diet.

## 2. Materials and Methods

Insects: Larvae of the three insect species (*E. kuehniella*, *T. molitor* and *H. illucens*) were reared with different diets, collected, and pre-processed (separated from the substrate residues, homogenized, lyophilized, and stored at −20 °C). Chemical compositions of the diets and growth results of the larvae are illustrated in Table 1. Two-liter aerated plastic containers (polypropylene) were prepared with 453, 93 and 192 first-instar larvae and 910, 223 and 410 g of substrate for *E. kuehniella*, *T. molitor* and *H. illucens*, respectively. For each species, the ratio of larvae to substrate was kept between 0.4 and 0.5. Nine replicates were made for each combination of byproduct and insect species, including their standard diets. The used larvae did not endure fasting prior to analysis, to mimic industrial production.

**Larvae pretreatment:** At the end of the growing period, larvae were collected, separated from the substrate residues by sieving, and stored at −80 °C. Prior to analyses, larvae were homogenized with a blender-mixer R401 (Robot Coupe, Isleworth, UK) in the presence of dry ice. Samples were lyophilized (Lyomicron 55, Coolvacuum Technologies, Barcelona, Spain), packed in multilayer (Al-PE) flexible bags under vacuum, and stored at −20 °C. One aliquot was used for fatty acid and sterol determination. 

**Fatty acid profile:** Fatty acid profiles were assessed using a method reported in a previous study [25]. In brief, samples (250 mg) were extracted with a mixture of chloroform:methanol (2:1, *v*/*v*), derivatized with a mixture of toluene and HCl 3 N in methanol (1:4, *v*/*v*) at 80 °C for 1 h, and added with NaCl 10% in water and hexane (10:3, *v*/*v*). Fatty acid methyl esters were recovered in the organic phase and then separated on a gas chromatograph Agilent 6890 Series II (Hewlett Packard SA, Barcelona, Spain) equipped with a capillary column DB23 (30 m × 0.25 mm i.d., 0.25 μm; Agilent, Santa Clara, CA, USA), a split–splitless injector, and a flame ionization detector. An initial oven temperature of 80 °C was used, with a gradient of 12 °C/min up to 140 °C, a gradient of 1.5 °C/min up to 190 °C, a gradient of 1.0 °C/min up to 200 °C, a gradient of 1.5 °C/min up to 205 °C, and a gradient of 3.0 °C/min up to 210 °C. Hydrogen was used as a carrier at a flow of 1.2 mL/min. Identification of single methyl esters were performed through comparing retention times of the peaks with those of pure standards (capric, lauric, tridecylic, myristic, palmitic, hypogeic, palmitoleic, margaric, stearic, oleic, linoleic, alpha linolenic, arachidonic, and eicosapentaenoic; Sigma Aldrich, MO, USA), whereas quantification was carried out using tripentadecanoin (Merck KGaA, Darmstadt, Germany) as an internal standard. The analyses were carried out in triplicates. Limits of detection (LOD) were estimated on the basis of the concentration corresponding to a signal-to-noise ratio (S:N) of 3.

**Sterols:** The lyophilized insects (250 mg) were hydrolyzed in Soxcap (FOSS IBERIA, S.A., Barcelona, Spain) with 4 N HCl for 5 h. Subsequently, an extraction was carried out with 350 mL of hexane: diethyl ether (2:1, *v*/*v*). The internal standard (5α-cholestan-3β-ol) was added to the evaporated extract and saponification was carried out using 8 mL of 9 N KOH for 3 h at 80 °C. For the extraction of the unsaponifiable fraction, 3 aliquots of 12 mL of hexane: diethyl ether (2:1) and 4 mL of ethanol were added. The organic fractions of the 3 extractions were dried and then derivatized with 50 μL of silanizing solution (Silylating mixture I according to Sweeley, Sigma Aldrich, MO, USA) for 1 h at 80 °C. The derivatized sample was dried with a nitrogen flow, resuspended with 1 mL isooctane:2-propanol and injected into the chromatographic equipment. Chromatographic analysis was carried out with a CP-3800 gas chromatograph (Varian Inova 500, Varian Inc., Palo Alto, CA, USA) equipped with a DB-5MS column (length: 30 m, diameter: 0.250 mm diameter, film thickness: 0.25 μm, Agilent Technologies, Santa Clara, CA, USA). An initial oven temperature of 80 °C was used, with a gradient of 10 °C/min up to 160 °C, a gradient of 5 °C/min up to 250 °C and a gradient of 1 °C/min up to 285 °C. Identifications of single sterols were performed through comparing retention times of the peaks with those of pure standards (cholesterol, campesterol, stigmasterol, β-sitosterol, and stigmastanol; Sigma Aldrich, MO, USA), whereas quantification was performed on the basis of the response of the internal standard. The analyses were carried out in triplicate. Limits of detection (LOD) were estimated on the basis of the concentration corresponding to a signal-to-noise ratio (S:N) of 3.

**Statistical analysis**: Fatty acid and sterol compositions were assessed in triplicate and data were expressed as means ± standard deviations (SD). The normality of data distribution was first verified through the Kolmogorov–Smirnov test and rejected. The Kruskal–Wallis test was performed to verify the diet composition on fatty acids, sterols, and lipid indices of *H. illucens* and *T. molitor*, followed by post hoc testing using Dunn’s multiple comparisons. A Mann–Whitney test was performed for *E. kuehniella*. These tests were performed at a significance level of α = 0.05. Finally, a principal component analysis (PCA) was performed to verify the effect of insect species on the compositional parameters. All the statistical analyses were performed using IBM SPSS 24 statistical software (SPSS Inc., Chicago, IL, USA).

## 3. Results and Discussion

In the present study, the larvae had not been fasted; therefore, the results presented reflect both insect fatty acid and sterol compositions as well as any food/diet remaining in the gastrointestinal tract of the insect.

### 3.1. Effect of Diet Composition on Fatty Acids Profiles

#### 3.1.1. *Hermetia illucens*

Table 2 reports the percentages and concentrations of 13 fatty acids quantified in *H. illucens*. Lauric acid was the most abundant, followed by palmitic acid and linoleic acid, in agreement with previous studies [11,22,29]. Our results are also consistent with previous publications reporting high levels of lauric acid in the lipidic fraction of *H. illucens* larvae [12]. Spranghers [30] suggested that *H. illucens* synthesizes lauric acid from carbohydrates in the substrate. In a recent study, it was found that rearing *H. illucens* on okara resulted in a drastic reduction in lauric acid (17.6% of total fatty acids), because okara has poor levels of carbohydrates (starch and sugars) required for lauric acid synthesis [31]. In fact, carbohydrates are a source of acetyl-CoA, which is necessary for the biosynthesis of fatty acids. This suggests that a high-carbohydrate diet could result in high acetyl-CoA production, and therefore high lauric acid levels [32].

The high content of lauric acid in the lipid fraction of *H. illucens* larvae was similar to that in coconut oil (~45–53%) [33]. Even though high lauric acid levels contribute to the increase in the percentage of saturated fatty acids, this fatty acid is of interest for humans and animals due its antimicrobial properties [34]. Lauric acid was reported as source of energy for fish feeding, resulting in a reduction in lipids in the liver [35]. Indeed, lauric acid is among the medium-chain fatty acids that can modulate intestinal health by regulating the level of IL-6 and TNF-α, and might contribute to appetite reduction [36].

Overall, irrespective of the type of diet, the concentration of total fatty acids in *H. illucens* larvae was dominated by saturated fatty acids (SFAs; 74%), followed by monounsaturated fatty acids (MUFA; up to 12%) and polyunsaturated fatty acids (PUFA; up to 14%). In previous studies, regardless of the diet (fish, bread, or food waste), similar ranges of variability (i.e., SFA (up to 76%), MUFA (up to 32%) and PUFA (up to 23%)) were reported [11,12]. The SFA:UFA ratio ranged from 2.85 to 3.86, exceeding the values recommended for a human healthy diet (<0.5) in all the treatments [37], whereas the ω6:ω3 was higher than indicated by the current nutritional recommendations (<4.0) [38], except in the case of the diet based on apricots. 

Generally, larvae obtained in this work contained linoleic acid (4.44–12.7%) levels comparable to larvae reared on chicken feed (14.7%), but lower than those reared on okara (28.4%), maize distillers (24.3%) fish offal (25.1%), brown algae (24%) or hemp seeds (26%) [11,31,39,40]. Oleic or linoleic were reported to be derived from biosynthesis pathways and diet accumulation, unlike lauric acid (exclusively synthesized) [32]. Diets including by-products did not significantly affect the total fatty acid (mg/100 mg) content, but produced significant effects on the fatty acid profiles. Compared to the control, the diet based on fresh apricots (AP-R) resulted in the accumulation of the highest amount of fatty acids (i.e., capric, lauric, palmitoleic, margaric and stearic), but lowered the concentration of the linoleic acid. Notably, arachidonic acid and EPA were found only in larvae fed this diet. In a previous study, EPA was found in insects reared on fish (1.7%), food waste (0.5%), and mussels (2%), but it was not found in those reared on bread [12]. These changes decreased the level of polyunsaturated fatty acids (PUFA) and the ω6:ω3 ratio and increased the SFA:UFA ratio compared to the control diet, with a global negative effect from a nutritional point of view (increasing the risk of cardiovascular disease, cancer, and inflammatory and autoimmune diseases) [38]. 

The larvae fed with pellet brewer’s spent grain, brewer’s spent yeast and feed mill by-products (Mixture Hi1) showed significantly higher amounts of palmitic acid and lower levels of linoleic and alpha-linolenic acids compared to larvae reared on the control diet. However, as a global effect, the SFA:UFA ratio was not significantly different from in the control samples.

Therefore, under our conditions, the diets including agroindustry by-products did not significantly modify the level of SFA but reduced the overall content of UFA. This finding supports the hypothesis that SFA and MUFA are mainly synthesized by this insect species [12], whereas PUFAs are most likely obtained from the substrate, as reported in the case of supplementation with seaweed and oil seeds [11,12].

#### 3.1.2. *Tenebrio molitor*

Eleven fatty acids were quantified in the larval biomass of *T. molitor* (Table 3). The lipid fractions of these biomasses were characterized, regardless of the diet composition, by high amounts of oleic, linoleic and palmitic acids, in good agreement with previous findings [41]. The most prevalent SFAs were palmitic acid, myristic acid, and stearic acid, consistent with prior studies [42,43]. Van Broekhoven et al. [19] reported the presence of EPA in *T. molitor* larvae reared on a diet with a high protein/low starch substrate; however, we did not detect EPA or DHA, in concordance with the results of Rumpold and Schlüter [44]. Overall, the total fatty acid content varied between 31.89 mg/100 g (DM) and 37.89 mg/100 g (DM), within the same range as reported elsewhere [44].

The diet based on brewer’s spent grain, brewer’s spent yeast and feed mill by-products (Mixture *Tm1*) had a limited impact on the fatty acid profile, increasing only the concentration of oleic and palmitoleic acids, as well as the MUFA, compared to the control diet. Compared to the control treatment, the diet based on feed mill by-products (FM) exhibited higher concentrations of oleic and linoleic acids together with high contents of total fatty acids, MUFA, PUFA and ω6 fatty acids. 

The level of ω3 fatty acids was quite low, irrespective of the type of diet, whereas the ω6 fatty acids and the ω6:ω3 ratio were slightly increased in the “Feed mill by-products” (FM) diet compared the “Control” diet. The ω6:ω3 ratio was always greater than 50, higher than in other studies reported in the literature [19,41,45], and significantly higher than 4.0, which is the suggested optimal value in food fats [46]. 

On the other hand, the SFA:UFA ratio was always lower than 0.5; these values are within the same range of previous studies on *T. molitor* reared on wheat, oat flour and bread [39,41], and indicate that the fat of all the biomasses of *T. molitor* had a proportion of saturated and unsaturated fatty acids suitable for human consumption. 

Even so, our outcomes seem to indicate that the fatty acid profile of *T. molitor* is significantly influenced by changes in the diet composition, consistent with previous studies which were carried out with different diets, including chia seeds, flax seeds, oat or wheat flours or vegetables [39,41,47].

#### 3.1.3. *Ephestia kuehniella*

In *E. kuehniella* larvae, seven fatty acids were identified, with oleic, palmitic and linoleic acid being the most abundant, regardless of the diet (Table 4). To the best of our knowledge, this is the first time that the fatty acid profile of *E. kuehniella* has been described.

In this species, the use of a diet based on by-products did not modify the total fatty acid content but had a relevant impact on the fatty acids profile. The diets based on “Feed mill by-products” and “Brewer’s spent yeast” significantly increased the concentration of myristic, palmitic, stearic, linoleic and alpha-linolenic acids, and reduced the level of oleic acid compared to the control diet. Therefore, the SFA, MUFA, UFA and PUFA were also affected. Diets based on by-product mixtures increased SFA, PUFA and both ω3 and ω6, but decreased MUFA. The overall effect was a decrease in the ω6:ω3 ratio and an increase in the SFA:UFA ratio, which was still around 0.5, indicating that the fat fraction of these biomasses could be interesting for both human and animal nutrition. 

### 3.2. Differences in Fatty Acid Profiles Between Insect Species

**Figure 1:**
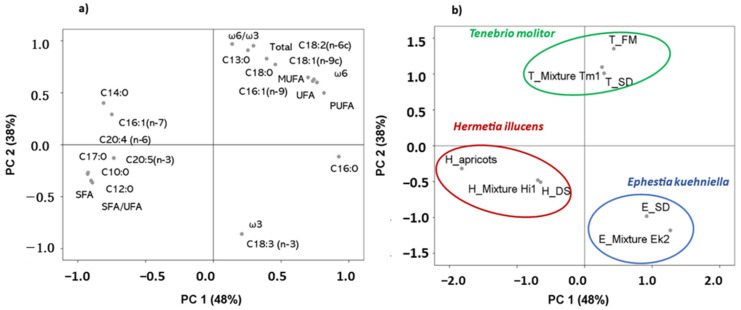
Scattering the data of fatty acids and lipid indices by the first two principal components (PC1 and PC2): analysis of different insect species fed different diets. (**a**) Biplot of the first two components created considering fatty acids and lipid indices; (**b**) rotated principal scores of insect species and their corresponding diets projected onto the first two principal components.

A principal component analysis of fatty acids was performed to better describe the modifications highlighted in all species simultaneously (Figure 1). The total accumulative variance from the first two principal components accounted for 86% of the total variance, where the first component accounted for 48% and the second component accounted for 38%. The first component was explained as a function of the major part of the fatty acids (i.e., margaric, palmitic, lauric, capric, myristic, linoleic, oleic, EPA, arachidonic, hypogeic, and palmitoleic) and lipid indices (SFA:UFA, SFA, PUFA, ω6, MUFA, and UFA), whereas the second component was expressed as a function of ω6:ω3, tridecylic, ω3, alpha linolenic, stearic and the total contents of fatty acids (Figure 1a). The projection of the different diets of each insect on the factorial space created by the fatty acid contents, and the lipid indices are illustrated in Figure 1b. Through PCA, a clear discrimination was observed as a function of insect species, whereas no clustering was observed as a function of diet composition. This emphasizes the fact that fatty acid composition is related more to insect species rather than diet composition. Further studies could focus on wider media formulations to investigate the potential of diet composition in tailoring the final outcomes of biomass composition.

The overlapping of both Figure 1a,b enabled identification of the parameters characterizing each species:

- *H. illucens*: margaric, capric, lauric, EPA, SFA, and SFA:UFA;
- *T. molitor:* ω6:ω3, tridecylic, stearic, total fatty acids;
- *E. kuehniella:* ω3, and alpha linolenic.

### 3.3. Effect of Diet Composition on Sterols Profiles

Insects lack the ability to synthesize sterols de novo due to the deficiency of enzymes for cellular synthesis; therefore, they generally obtain sterols from their diets [48]. Table 5 summarizes the phytosterol and cholesterol profiles as functions of diet and insect species. In all species, cholesterol, campesterol, stigmasterol, β-sitosterol, and stigmastanol were identified. The use of different by-products in the diet caused dramatic changes in the sterol profiles of the three larvae species. 

In the case of *H. illucens*, β-sitosterol was the main sterol identified followed, by campesterol, consistent with previous studies [11,49,50]. Compared to the control diet, the diet based on fresh apricots (AP-R) increased cholesterol and stigmasterol and decreased campesterol, while no β-sitosterol or stigmastanol were detected. The larvae fed with pellet “Brewer’s spent grain, Brewer’s spent yeast and feed mill by-products” (Mixture Hi1) contained more cholesterol and stigmasterol, but less campesterol and stigmastanol than those reared on the “Control” diet.

For *T. molitor* larvae, cholesterol, stigmasterol, β-sitosterol and stigmastanol were significantly impacted by diet composition. The “Feed mill by-products” diet (FM) resulted in the highest cholesterol and stigmasterol levels, whereas the “Mixture *Tm1*” diet resulted in the highest stigmastanol. This result is in concordance with previous studies [51,52]. Compared to the control diet, no significant differences were found in campesterol levels. 

In *E. kuehniella*, the “Mixture Ek2” diet induced higher cholesterol and stigmasterol, but lower stigmastanol compared to the control diet. However, the larvae fed with the “Mixture Ek2” diet had similar amounts of β-sitosterol and campesterol compared to the “Control” diet. To the best of our knowledge, this is the first time that the sterol profile of *E. kuehniella* has been described. 

### 3.4. Differences in Fatty Sterol Profiles between Insects Species

**Figure 2:**
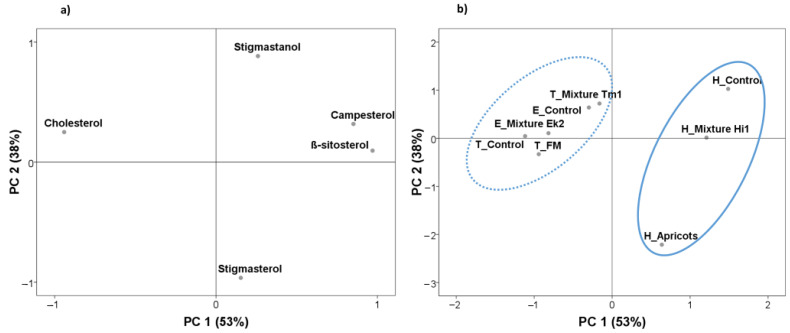
Scattering the data of sterols by the first two principal components (PC1 and PC2): analysis of different insect species fed different diets. (**a**) Biplot of the first two components created considering sterols; (**b**) rotated principal scores of insect species and their corresponding diets projected onto the first two principal components.

Figure 2 shows the results of principal component analysis of sterols. The total accumulative variance from the first two principal components accounted for 91% of the total variance, where the first components accounted for 53% and the second for 38% (Figure 2a). The first component was expressed as a function of β-sitosterol, cholesterol and campesterol, whereas the second was described in terms of stigmasterol and stigmastanol. The projection of the diet composition and insects showed two groups on both sides of the first principal components (Figure 2b). *H. illucens* was located on the positive side due to its high campesterol and β-sitosterol levels, whereas *T. molitor* and *E. kuehniella* were located on the negative side due to their high cholesterol and low campesterol and β-sitosterol levels. No clear separation was found as a function of diet composition for *E. kuehniella* and *T. molitor*.

## 4. Conclusions

In the present study, diets formulated with the inclusion of agro-industrial by-products enabled growth of the larval biomasses and caused significant changes to the fatty acid compositions of the three insect species, but did not modify the typical overall characteristics of the lipid fraction of each species. Multivariate analysis (PCA) based on fatty acid and lipid indices clustered each species separately regardless of diet composition. Sterol profiles were influenced by the inclusion of by-products in the diet; furthermore, a clear discrimination was found between *H. illucens* (low cholesterol and high campesterol and β-sitosterol contents), and *T. molitor* and *E. kuehniella* (high cholesterol and low campesterol contents). Notably, the fatty acid and sterol profiles of *E. kuehniella* have been studied for the first time, enabling new insights to the nutritional composition of this species and its potential use as food or feed. Results from this study show that insects can provide a suitable bioconversion of agro-industrial by-products to produce a lipid-rich biomass for feed and food applications.

## Figures and Tables

**Table 1:** Description of the diets included in this study (main components as described in Riudavets et al. [25]).

Treatments	Diet Description	Diet Composition			Mortality (%)	Weight (mg)	Development Time (Weeks)	
		Moisture (%)	Total Fat (% Dry Matter)	Total Protein (% Dry Matter)			Pupae	Adult
*H. illucens*								
Control [26]	24.4% wheat bran, 14.6% rabbit feed (Cryspy Muesli, Versele-Laga), 2.4% yeast, and 58.6%, water	61.2	1.8	7.5	21.6 ± 6.6	225.7 ± 10.3	3	5
AP-R	100% apricots (raw)	88.5	0.2	0.6	83.9 ± 2.9	309.9 ± 31.8	3	8
Mixture *Hi*1	25% brewer’s spent grain (dried), 12.5% feed mill by-products and 62.5% brewer’s spent yeast (raw)	59.1	3.0	9.6	32.1 ± 7.7	292.0 ± 11.9	3	5
*T. molitor*								
Control [27]	48.5% whole flour wheat, 36.7% wheat bran, and 14.7% pet food (Ultima dog food with chicken, Affinity Petcare)	12.1	6.0	17.2	8.0 ± 5.8	156.5 ± 4.4	11	13
FM	100% feed mill by-products (broken cereal grains)	8.7	2.9	11.3	39.7 ± 3.3	142.4 ± 5.5	16	18
Mixture *Tm*1	36.8% brewer’s spent grain (dried), 52.5% feed mill by-products: and 10.5% brewer’s spent yeast (dried)	7.3	4.9	19.3	22.8 ± 2.3	140.1 ± 12.1	11	13
*E. kuehniella*								
Control [28]	67% whole wheat flour and 33%, commercial yeast	11.4	2.6	16.4	60.4 ± 2.4	43.5 ± 2.5	3	4
Mixture *Ek*2	67% feed mill by-products and 33%, brewer’s spent yeast (dried)	6.5	2.7	22.4	38.4 ± 7.5	18.3 ± 2.5	6	7

**Table 2:** Percentages and concentration (means ± standard deviation) of fatty acids in *H. illucens* larvae as a function of the diet. Results are expressed per dry matter (DM). Means within a row with different letters differ significantly (* *p* ≤ 0.05; ** *p* ≤ 0.01; *** *p* ≤ 0.001).

Fatty Acids and Indices	Control	Mixture Hi1	AP-R	Significance
Capric [C10:0] (%)	0.57 ± 0.11 a	0.52 ± 0.26 a	1.36 ± 0.06 b	**
Lauric [C12:0] (%)	50.83 ± 2.74 a	49.86 ± 2.43 a	55.39 ± 0.62 b	*
Tridecylic |C13:0] (%)	*n.d.*	*n.d.*	*n.d.*	
Myristic [C14:0] (%)	8.74 ± 0.32	8.34 ± 0.88	7.50 ± 0.14	ns
Palmitic [C16:0] (%)	11.92 ± 0.46 a	13.96 ± 0.75 b	11.99 ± 0.21 a	**
Hypogeic [C16:1(n-9c)] (%)	0.80 ± 0.24 b	0.46 ± 0.07 a	0.19 ± 0.00 a	**
Palmitoleic [C16:1(n-7c)] (%)	1.71 ± 0.14 a	2.00 ± 0.35 a	4.35 ± 0.07 b	***
Margaric [C17:0] (%)	0.18 ± 0.03 a	0.22 ± 0.09 a	0.38 ± 0.01 b	**
Stearic [C18:0] (%)	1.61 ± 0.08 a	1.89 ± 0.34 ab	2.18 ± 0.05 b	*
Oleic [C18:1(n-9c)] (%)	9.17 ± 0.69	11.35 ± 1.07	9.64 ± 0.71	ns
Linoleic [C18:2(n-6c)] (%)	12.7 ± 1.74 c	10.47 ± 0.33 b	4.44 ± 0.09 a	*
Alpha linolenic [C18:3(n-3c)] (%)	1.77 ± 0.08 b	0.93 ± 0.09 a	1.85 ± 0.06 b	***
Arachidonic [C20:4(n-6c)] (%)	*n.d.*	*n.d.*	0.17 ± 0.00	
Eicosapentaenoic (EPA) [C20:5(n-3c)] (%)	*n.d.*	*n.d.*	0.57 ± 0.02	
Capric [C10:0] (mg/100 mg)	0.16 ± 0.04 a	0.13 ± 0.07 a	0.37 ± 0.02 b	**
Lauric [C12:0] (mg/100 mg)	13.83 ± 1.81 a	11.84 ± 0.9 a	15.07 ± 0.31 b	*
Tridecylic |C13:0] (mg/100 mg)	*n.d.*	*n.d.*	*n.d.*	
Myristic [C14:0] (mg/100 mg)	2.38 ± 0.26	1.98 ± 0.23	2.04 ± 0.01	ns
Palmitic [C16:0] (mg/100 mg)	3.23 ± 0.12 a	3.33 ± 0.45 b	3.26 ± 0.02 a	**
Hypogeic [C16:1(n-9c)] (mg/100 mg)	0.21 ± 0.05 b	0.11 ± 0.01 a	0.05 ± 0.00 a	**
Palmitoleic [C16:1(n-7c)] (mg/100 mg)	0.46 ± 0.01 a	0.47 ± 0.04 a	1.18 ± 0.01 b	***
Margaric [C17:0] (mg/100 mg)	0.05 ± 0.00 a	0.05 ± 0.04 a	0.10 ± 0.00 b	**
Stearic [C18:0] (mg/100 mg)	0.44 ± 0.05 a	0.45 ± 0.11 ab	0.59 ± 0.00 b	*
Oleic [C18:1(n-9c)] (mg/100 mg)	2.48 ± 0.04	2.71 ± 0.43	2.63 ± 0.24	ns
Linoleic [C18:2(n-6c)] (mg/100 mg)	3.43 ± 0.28 c	2.49 ± 0.28 b	1.21 ± 0.04 a	*
Alpha linolenic [C18:3(n-3c)] (mg/100 mg)	0.48 ± 0.03 b	0.22 ± 0.03 a	0.50 ± 0.01 b	***
Arachidonic [C20:4(n-6c)] (mg/100 mg)	*n.d.*	*n.d.*	0.05 ± 0.00	
Eicosapentaenoic (EPA) [C20:5(n-3c)] (mg/100 mg)	*n.d.*	*n.d.*	0.15 ± 0.00	
Sum of fatty acids (mg/100 mg)	27.15 ± 2.03	23.78 ± 2.07	27.21 ± 0.53	ns
SFA (mg/100 mg)	20.08 ± 2.27	17.77 ± 1.46	21.44 ± 0.33	ns
MUFA (mg/100 mg)	3.16 ± 0.06 a	3.29 ± 0.38 a	3.86 ± 0.25 b	*
PUFA (mg/100 mg)	3.90 ± 0.27 c	2.72 ± 0.31 b	1.71 ± 0.04 a	***
ω6 (mg/100 mg)	3.43 ± 0.28 c	2.49 ± 0.30 b	1.21 ± 0.04 a	***
ω3 (mg/100 mg)	0.48 ± 0.03 b	0.22 ± 0.03 a	0.5 ± 0.01 b	***
ω6:ω3	7.17 ± 0.74 b	11.33 ± 1.69 c	2.4 ± 0.05 a	***
UFA (mg/100 mg)	7.06 ± 0.31 b	6.00 ± 0.67 a	5.57 ± 0.29 a	*
SFA:UFA	2.85 ± 0.43 a	2.97 ± 0.17 a	3.86 ± 0.18 b	*

SFA, sum of saturated fatty acids; MUFA, sum of monounsaturated fatty acids; PUFA, sum of polyunsaturated fatty acids; ω6, sum of omega-6 polyunsaturated fatty acids; ω3, sum of omega-3 polyunsaturated fatty acids; UFA, sum of monounsaturated fatty acids + polyunsaturated fatty acids. *n.d.*, under the limit of detection (<0.01 mg/100 mg); ns, not significant; * *p* ≤ 0.05; ** *p* ≤ 0.01; *** *p* ≤ 0.001.

**Table 3:** Percentages and concentration (means ± standard deviation) of fatty acids in *T. molitor* larvae as a function of the diet. Results are expressed per dry matter (DM). Means within a row with different letters differ significantly.

Fatty Acids and Indices	Control	FM	Mixture *Tm*1	Significance
Capric [C10:0] (%)	0.02 ± 0.01	0.02 ± 0.01	0.02 ± 0.01	ns
Lauric [C12:0] (%)	0.46 ± 0.03 b	0.37 ± 0.02 a	0.41 ± 0.06 b	**
Tridecylic [C13:0] (%)	0.05 ± 0.00	0.03 ± 0.00	0.04 ± 0.01	ns
Myristic [C14:0] (%)	4.61 ± 0.11 b	4.19 ± 0.14 a	4.68 ± 0.39 b	*
Palmitic [C16:0] (%)	18.20 ± 0.48	16.12 ± 0.48	17.40 ± 0.82	ns
Hypogeic [C16:1(n-9c)] (%)	1.69 ± 0.07 b	1.54 ± 0.05a	1.53 ± 0.15 a	**
Palmitoleic [C16:1(n-7c)] (%)	1.68 ± 0.01	1.60 ± 0.03	2.05 ± 0.15	ns
Margaric [C17:0] (%)	*n.d.*	*n.d.*	*n.d.*	
Stearic [C18:0] (%)	3.58 ± 0.12 a	3.64 ± 0.18 b	3.38 ± 0.57 b	**
Oleic [C18:1(n-9c)] (%)	49.63 ± 0.50 a	52.37 ± 0.60 b	51.95 ± 0.94 b	***
Linoleic [C18:2(n-6c)] (%)	19.72 ± 0.11 b	19.82 ± 0.17 b	18.21 ± 0.28 a	**
Alpha linolenic [C18:3(n-3c)] (%)	0.36 ± 0.02	0.29 ± 0.01	0.34 ± 0.02	ns
Arachidonic [C20:4(n-6c)] (%)	*n.d.*	*n.d.*	*n.d.*	
Eicosapentaenoic (EPA) [C20:5(n-3c)] (%)	*n.d.*	*n.d.*	*n.d.*	
Capric [C10:0] (mg/100 mg)	0.01 ± 0.00	0.01 ± 0.00	0.01 ± 0.00	ns
Lauric [C12:0] (mg/100 mg)	0.15 ± 0.02	0.14 ± 0.01	0.14 ± 0.02	ns
Tridecylic [C13:0] (mg/100 mg)	0.02 ± 0.00	0.01 ± 0.00	0.01 ± 0.00	ns
Myristic [C14:0] (mg/100 mg)	1.47 ± 0.11	1.59 ± 0.16	1.61 ± 0.19	ns
Palmitic [C16:0] (mg/100 mg)	5.80 ± 0.17	6.11 ± 0.60	5.98 ± 0.30	ns
Hypogeic [C16:1(n-9c)] (mg/100 mg)	0.54 ± 0.04	0.58 ± 0.02	0.53 ± 0.07	ns
Palmitoleic [C16:1(n-7c)] (mg/100 mg)	0.53 ± 0.03 a	0.61 ± 0.05 ab	0.71 ± 0.07 b	*
Margaric [C17:0] (mg/100 mg)	*n.d.*	*n.d.*	*n.d.*	
Stearic [C18:0] (mg/100 mg)	1.14 ± 0.02	1.38 ± 0.10	1.16 ± 0.16	ns
Oleic [C18:1(n-9c)] (mg/100 mg)	15.83 ± 0.92 a	19.82 ± 1.25 b	17.85 ± 0.67 b	**
Linoleic [C18:2(n-6c)] (mg/100 mg)	6.29 ± 0.32 a	7.50 ± 0.56 b	6.26 ± 0.30 a	*
Alpha linolenic [C18:3(n-3c)] (mg/100 mg)	0.11 ± 0.01	0.11 ± 0.00	0.12 ± 0.01	ns
Arachidonic [C20:4(n-6c)] (mg/100 mg)	*n.d.*	*n.d.*	*n.d.*	
Eicosapentaenoic (EPA) [C20:5(n-3c)] (mg/100 mg)	*n.d.*	*n.d.*	*n.d.*	
Sum of fatty acids (mg/100 mg)	31.89 ± 1.60 a	37.86 ± 2.71 b	34.36 ± 1.21 ab	*
SFA (mg/100 mg)	8.58 ± 0.31	9.24 ± 0.84	8.91 ± 0.35	ns
MUFA (mg/100 mg)	16.91 ± 0.98 a	21.01 ± 1.32 b	19.08 ± 0.81 b	**
PUFA (mg/100 mg)	6.41 ± 0.33 a	7.62 ± 0.56 b	6.38 ± 0.30 a	*
ω6 (mg/100 mg)	6.29 ± 0.32 a	7.50 ± 0.56 b	6.26 ± 0.30 a	*
ω3 (mg/100 mg)	0.11 ± 0.01	0.11 ± 0.00	0.12 ± 0.01	ns
ω6:ω3	54.91 ± 2.82 a	66.74 ± 2.65 b	53.97 ± 1.50 a	**
UFA (mg/100 mg)	23.31 ± 1.30 a	28.62 ± 1.87 b	25.46 ± 1.06 a	*
SFA:UFA	0.37 ± 0.01 b	0.32 ± 0.01 a	0.35 ± 0.02 b	*

SFA, sum of saturated fatty acids; MUFA, sum of monounsaturated fatty acids; PUFA, sum of polyunsaturated fatty acids; ω6, sum of omega-6 polyunsaturated fatty acids; ω3, sum of omega-3 polyunsaturated fatty acids; UFA, sum of monounsaturated fatty acids + polyunsaturated fatty acids. *n.d.*, under the limit of detection (<0.01 mg/100 mg); ns, not significant; * *p* ≤ 0.05; ** *p* ≤ 0.01; *** *p* ≤ 0.001.

**Table 4:** Percentages and concentration (means ± standard deviation) of fatty acids in *E. kuehniella* larvae as a function of the diet. Results are expressed per dry matter (DM).

Fatty Acids and Indices	Control	Mixture Ek2	Significance
Capric [C10:0] (%)	*n.d.*	*n.d.*	
Lauric [C12:0] (%)	*n.d.*	*n.d.*	
Tridecylic [C13:0] (%)	*n.d.*	*n.d.*	
Myristic [C14:0] (%)	0.19 ± 0.01	0.32 ± 0.04	**
Palmitic [C16:0] (%)	29.02 ± 0.12	30.34 ± 0.54	*
Hypogeic [C16:1(n-9c)] (%)	*n.d.*	*n.d.*	
Palmitoleic [C16:1(n-7c)] (%)	1.24 ± 0.03	1.21 ± 0.06	ns
Margaric [C17:0] (%)	*n.d.*	*n.d.*	
Stearic [C18:0] (%)	1.69 ± 0.05	3.44 ± 0.49	**
Oleic [C18:1(n-9c)] (%)	51.42 ± 0.53	40.68 ± 2.47	**
Linoleic [C18:2(n-6c)] (%)	14.43 ± 0.28	20.72 ± 1.25	**
Alpha linolenic [C18:3(n-3c)] (%)	2.01 ± 0.10	3.29 ± 0.24	**
Arachidonic [C20:4(n-6c)] (%)	*n.d.*	*n.d.*	
Eicosapentaenoic (EPA) [C20:5(n-3c)] (%)	*n.d.*	*n.d.*	
Capric [C10:0] (mg/100 mg)	*n.d.*	*n.d.*	
Lauric [C12:0] (mg/100 mg)	*n.d.*	*n.d.*	
Tridecylic C13:0 (mg/100 mg)	*n.d.*	*n.d.*	
Myristic [C14:0] (mg/100 mg)	0.05 ± 0.01	0.09 ± 0.01	**
Palmitic [C16:0] (mg/100 mg)	8.11 ± 0.47	8.65 ± 0.45	ns
Hypogeic [C16:1(n-9c)] (mg/100 mg)	*n.d.*	*n.d.*	
Palmitoleic [C16:1(n-7c)] (mg/100 mg)	0.35 ± 0.02	0.34 ± 0.04	ns
Margaric [C17:0] (mg/100 mg)	*n.d.*	*n.d.*	
Stearic [C18:0] (mg/100 mg)	0.47 ± 0.04	0.98 ± 0.09	**
Oleic [C18:1(n-9c)] (mg/100 mg)	14.35 ± 0.73	11.63 ± 1.45	*
Linoleic [C18:2(n-6c)] (mg/100 mg)	4.03 ± 0.26	5.90 ± 0.24	**
Alpha linolenic [C18:3(n-3c)] (mg/100 mg)	0.56 ± 0.05	0.94 ± 0.06	**
Arachidonic [C20:4(n-6c)] (mg/100 mg)	*n.d.*	*n.d.*	
Eicosapentaenoic (EPA) [C20:5(n-3c)] (mg/100 mg)	*n.d.*	*n.d.*	
Sum of fatty acids (mg/100 mg)	27.92 ± 1.54	28.53 ± 1.95	ns
SFA (mg/100 mg)	8.63 ± 0.51	9.72 ± 0.42	*
MUFA (mg/100 mg)	14.70 ± 0.75	11.98 ± 1.49	*
PUFA (mg/100 mg)	4.59 ± 0.31	6.83 ± 0.30	**
ω6 (mg/100 mg)	4.03 ± 0.26	5.90 ± 0.24	**
ω3 (mg/100 mg)	0.56 ± 0.05	0.94 ± 0.06	**
ω6:ω3	7.21 ± 0.02	6.03 ± 0.13	**
UFA (mg/100 mg)	19.29 ± 1.03	18.81 ± 1.56	ns
SFA:UFA	0.45 ± 0.00	0.52 ± 0.02	***

SFA, sum of saturated fatty acids; MUFA, sum of monounsaturated fatty acids; PUFA, sum of polyunsaturated fatty acids; ω6, sum of omega-6 polyunsaturated fatty acids; ω3, sum of omega-3 polyunsaturated fatty acids; UFA, sum of monounsaturated fatty acids + polyunsaturated fatty acids. *n.d.*, under the limit of detection (<0.01 mg/100 mg); ns, not significant; * *p* ≤ 0.05; ** *p* ≤ 0.01; *** *p* ≤ 0.001.

**Table 5:** Concentration of sterols (g/kg dry matter) in the three larvae species as a function of diet.

Sterols	*H. illucens*			*T. molitor*			*E. kuehniella*	
	Control	Mixture Hi1	Apricots	Control	FM	Mixture *Tm*1	Control	Mixture Ek2
Cholesterol	0.031 ± 0.004 a	0.225 ± 0.022 c	0.048 ± 0.008 b	1.037 ± 0.058 b	0.935 ± 0.048 b	0.646 ± 0.103 a	0.873 ± 0.15 a	1.13 ± 0.127 b
Campesterol	0.554 ± 0.054 c	0.478 ± 0.017 b	0.122 ± 0.0 4 a	0.078 ± 0.014	0.083 ± 0.020	0.073 ± 0.092	0.132 ± 0.012	0.136 ± 0.010
Stigmasterol	*n.d.*	0.043 ± 0.003 a	0.154 ± 0.010 b	0.018 ± 0.002 a	0.044 ± 0.003 b	0.029 ± 0.012 a	0.014 ± 0.006 a	0.035 ± 0.008 b
β-sitosterol	1.035 ± 0.090 b	1.017 ± 0.012 a	*n.d.*	0.168 ± 0.027 a	0.209 ± 0.027 b	0.171 ± 0.072 ab	0.383 ± 0.018	0.364 ± 0.028
Stigmastanol	0.113 ± 0.012 b	0.062 ± 0.023 a	*n.d.*	0.039 ± 0.006 a	0.034 ± 0.004 a	0.123 ± 0.008 b	0.091 ± 0.027 b	0.054 ± 0.016 a

*n.d.*, lower than the detection limit (0.01 mg/g dry matter). Values are shown as means ± standard deviation. Means within a row for each species with different letters differ significantly (*p* ≤ 0.05).

## Data Availability

Not applicable.
